# Participatory development of training videos for respiratory equipment

**DOI:** 10.2471/BLT.24.291816

**Published:** 2024-10-02

**Authors:** Joshua Tim, Bill Gentles, J Tobey Clark, Pryanka Relan, Marta Lado, Hui-Ling Lin, Michael Lipnick, Barun Kumar Rauniyar, Daniela Rodriguez Rodriguez, Adriana Velazquez Berumen

**Affiliations:** a Ministry of Health Botswana, Plot 54609, Amos Street, Government Enclave, PO Box 20172, Gaborone, Botswana.; b BT Medical Technology Consulting, Peterborough, Canada.; c WHO Collaborating Centre for Health Technology Management, Technical Services Partnership, University of Vermont, Burlington, United States of America (USA).; d Integrated Health Services Department, World Health Organization, Geneva, Switzerland.; e Partners in Health, Freetown, Sierra Leone.; f Chang Gung University, Tao-Yuan, Taiwan, China.; g WHO Collaborating Centre for Emergency, Critical and Operative Care, Department of Emergency Medicine, University of California at San Francisco, San Francisco, USA.; h Infectious Hazard Management Unit, WHO Regional Office for South-East Asia, New Delhi, India.; i Medical Devices and Diagnostics, World Health Organization, Geneva, Switzerland.

## Abstract

**Problem:**

During the coronavirus disease 2019 (COVID-19) pandemic, medical oxygen therapy was urgently needed for patients with hypoxaemia. Many low- and middle-income countries lacked the medical devices for oxygen therapy and experience in their use.

**Approach:**

In addition to providing medical devices for oxygen therapy for countries in need, the World Health Organization (WHO) and partners developed training videos to help local health workers select, use and maintain this equipment. Diverse health professionals, including engineers and clinicians from resource-constrained countries, collaborated in developing draft videos in their local settings. A production team refined these drafts and delivered the training videos through the platform OpenWHO.

**Local setting:**

OpenWHO is WHO’s free open-access platform providing courses for health workers and others. The courses, based on WHO’s scientific and operational guidance, can be easily adapted, contextualized and translated.

**Relevant changes:**

The production team refined the drafts into 32 training videos. More than 17 505 health workers participated in the OpenWHO course on COVID-19 respiratory equipment between 28 February 2022 and 30 November 2023. Participants were from 189 countries and 38% (6027/16 047) were from low- and lower-middle-income countries.

**Lessons learnt:**

Involving volunteer biomedical engineers and clinicians from low- and middle-income countries helped provide an appropriate training resource. WHO should continue to develop such training tools and offer them through OpenWHO, especially for emergencies.

## Résumé

**Problème:**

Lors de la pandémie de coronavirus en 2019 (COVID-19), les patients atteints d’hypoxémie ont eu besoin d’une oxygénothérapie médicale d’urgence. De nombreux pays à revenus faibles ou intermédiaires manquaient alors de dispositifs médicaux pour assurer cette oxygénothérapie et d’expérience dans leur utilisation.

**Approche:**

L’Organisation mondiale de la santé (OMS) et ses partenaires ont non seulement fourni des dispositifs médicaux d’oxygénothérapie aux pays qui en avaient besoin, mais ils ont également élaboré des vidéos de formation pour aider les professionnels de la santé locaux à sélectionner, utiliser et entretenir ce matériel. Différents professionnels de la santé, dont des ingénieurs et des cliniciens issus de pays aux ressources limitées, ont coopéré à l’élaboration de projets de vidéos dans leur environnement local. Une équipe de production a perfectionné ces projets et a diffusé les vidéos de formation par l’intermédiaire de la plateforme OpenWHO.

**Environnement local:**

OpenWHO est une plateforme gratuite et en accès libre de l’OMS qui propose des formations aux agents de santé et à d’autres personnes. Les formations, basées sur les orientations scientifiques et opérationnelles de l’OMS, peuvent être facilement adaptées, contextualisées et traduites.

**Changements significatifs:**

L’équipe de production a perfectionné les projets et a réalisé 32 vidéos de formation à partir de ces projets. Plus de 17 505 agents de santé ont participé à la formation OpenWHO sur les appareils respiratoires dans le cadre de la pandémie de COVID-19 entre le 28 février 2022 et le 30 novembre 2023. Les participants étaient originaires de 189 pays, et 38% d’entre eux (6027/16 047) provenaient de pays à revenus faibles ou intermédiaires de la tranche inférieure.

**Leçons tirées:**

La participation d’ingénieurs biomédicaux et de cliniciens volontaires issus de pays à revenus faibles ou intermédiaires a permis de fournir des ressources de formation appropriées. L’OMS devrait continuer à élaborer de tels outils de formation et les proposer sur la plateforme OpenWHO, en particulier pour les situations d’urgence.

## Resumen

**Situación:**

Durante la pandemia de la enfermedad por coronavirus de 2019 (COVID-19), se necesitó urgentemente oxigenoterapia médica para los pacientes con hipoxemia. Muchos países de ingresos bajos y medios carecían de los dispositivos médicos necesarios para la oxigenoterapia y de experiencia en su uso.

**Enfoque:**

Además de proporcionar dispositivos médicos para oxigenoterapia a los países necesitados, la Organización Mundial de la Salud (OMS) y sus asociados elaboraron vídeos de formación para ayudar al personal sanitario local a seleccionar, utilizar y mantener estos equipos. Varios profesionales sanitarios, entre ellos ingenieros y médicos de países con recursos limitados, colaboraron en la elaboración de los borradores de los vídeos en sus entornos locales. Un equipo de producción perfeccionó estos borradores y entregó los vídeos de formación a través de la plataforma OpenWHO.

**Marco regional:**

OpenWHO es la plataforma gratuita de libre acceso de la OMS que ofrece cursos para el personal sanitario y otras personas. Los cursos, basados en las orientaciones científicas y operativas de la OMS, pueden adaptarse, contextualizarse y traducirse fácilmente.

**Cambios importantes:**

El equipo de producción mejoró los borradores hasta convertirlos en 32 vídeos de formación. Más de 17 505 agentes de salud participaron en el curso OpenWHO sobre equipos respiratorios COVID-19 entre el 28 de febrero de 2022 y el 30 de noviembre de 2023. Los participantes procedían de 189 países y el 38% (6027/16 047) de países de ingresos bajos y medios bajos.

**Lecciones aprendidas:**

La participación de ingenieros biomédicos y médicos voluntarios de países de ingresos bajos y medios contribuyó a proporcionar un recurso de formación adecuado. La OMS debería seguir desarrollando estas herramientas de formación y ofrecerlas a través de OpenWHO, en especial para emergencias.

## ملخص

**المشكلة:**

أثناء جائحة مرض فيروس كورونا 2019 (كوفيد19)، كانت هناك حاجة شديدة للعلاج بالأكسجين الطبي للمرضى الذين يعانون من نقص التأكسج بالدم. وكانت العديد من الدول ذات الدخل المنخفض والدخل المتوسط في حاجة إلى الأجهزة الطبية للعلاج بالأكسجين فضلاً عن الخبرة في استخدامها.

**الأسلوب:**

بالإضافة إلى توفير الأجهزة الطبية اللازمة للعلاج بالأكسجين في الدول التي تحتاج إلى هذه الأجهزة، قامت منظمة الصحة العالمية (WHO) وشركاؤها بتطوير مقاطع فيديو تدريبية لمساعدة العاملين في قطاع الصحة المحلي في انتقاء هذه المعدات واستخدامها وصيانتها. تعاون العديد من المتخصصين الصحيين، بما في ذلك المهندسون والأطباء من الدول ذات الموارد المحدودة، في تطوير مقاطع فيديو أولية في بيئاتهم المحلية. قام فريق الإنتاج بتنقيح هذه المقاطع الأولية، وتسليم مقاطع التدريب النهائية من خلال منصة OpenWHO.

**المواقع المحلية:**

إن OpenWHO هي المنصة مفتوحة المصدر والمجانية لمنظمة الصحة العالمية، وهي تقدم دورات تدريبية للعاملين في القطاع الصحي وغيرهم. يمكن بسهولة تكييف الدورات التدريبية، المستندة إلى التوجيهات العلمية والتشغيلية لمنظمة الصحة العالمية، وصياغتها وترجمتها.

**التغيّرات ذات الصلة:**

قام فريق الإنتاج بتنقيح مقاطع الفيديو الأولية، وتحويلها إلى 32 مقطعًا من تدريبات الفيديو. شارك أكثر من 17505 من العاملين في القطاع الصحي في دورة OpenWHO التدريبية عن معدات التنفس الخاصة بكوفيد 19 في الفترة ما بين 28 فبراير/شباط 2022 و30 نوفمبر/تشرين ثاني 2023. وكان المشاركون من 189 دولة، بينما كان %38 منهم (6027/16047) من الدول ذات الدخل المنخفض، والدخل المتوسط المنخفض.

**الدروس المستفادة:**

إن اشتراك المهندسين والأطباء المتطوعين في مجال الطب الحيوي من الدول ذات الدخل المنخفض والدخل المتوسط، ساعد في توفير مورد تدريبي مناسب. يجب أن تستمر منظمة الصحة العالمية في تطوير مثل هذه الأدوات التدريبية، وطرحها من خلال منصة OpenWHO، وخاصة في حالات الطوارئ.

## 摘要

**问题:**

在新冠病毒 (COVID-19) 疫情期间，低氧血症患者急需接受医用氧疗。许多中低收入国家缺乏医用氧疗设备和相关使用经验。

**方法:**

除为有需要的国家提供医用氧疗设备外，世界卫生组织 (WHO) 及其合作伙伴还开发了培训视频，以帮助当地卫生工作者选择、使用和维护该设备。包括来自资源受限国家的生物医学工程师和临床医生在内的各种卫生专业人员合作，在当地开发了草拟培训视频。制作团队对这些草案进行了优化，并通过 OpenWHO 平台发布了这些培训视频。

**当地状况:**

OpenWHO 是 WHO 分享知识、为卫生工作者和类似人员提供线上课程的免费平台。这些课程以 WHO 的科学和操作指导为基础，很容易进行改编、情境化处理和翻译。

**相关变化:**

制作团队将优化后的草案剪辑成了 32 个培训视频。2022 年 2 月 28 日至 2023 年 11 月 30 日期间，有超过 17,505 名卫生工作者参加了 OpenWHO 上关于 COVID-19 呼吸设备的在线课程。参与者遍布 189 个国家，其中 38% (6027/16047) 来自中低收入国家。

**经验教训:**

让来自中低收入国家的志愿生物医学工程师和临床医生参与进来，有助于提供适用的培训资源。WHO 应继续致力于开发此类培训工具，并通过 OpenWHO 提供这些工具，特别是针对突发事件。

## Резюме

**Проблема:**

Во время пандемии коронавирусной инфекции 2019 г. (COVID-19) пациентам с гипоксемией срочно требовалась терапия медицинским кислородом. Во многих странах с низким и средним уровнем дохода отсутствуют медицинские изделия для кислородной терапии и опыт их использования.

**Подход:**

Помимо поставки медицинских изделий для кислородной терапии в нуждающиеся страны, Всемирная организация здравоохранения (ВОЗ) и ее партнеры разработали обучающие видеоматериалы, направленные на оказание помощи местным медицинским работникам в выборе, использовании и обслуживании таких изделий. Различные специалисты в области здравоохранения, включая инженеров и клиницистов из стран с ограниченными ресурсами, сотрудничали при разработке проектов видеороликов в своих регионах. Производственная группа доработала эти проекты и передала учебные видеоматериалы через платформу OpenWHO.

**Местные условия:**

OpenWHO – это бесплатная платформа ВОЗ с открытым доступом, на которой размещены курсы для работников здравоохранения и других специалистов. Курсы, основанные на научном и оперативном руководстве ВОЗ, могут быть легко адаптированы и переведены с учетом конкретных условий.

**Осуществленные перемены:**

Производственная группа доработала пробные версии до 32 учебных видеороликов. В период с 28 февраля 2022 года по 30 ноября 2023 года более 17 505 медицинских работников прошли курс OpenWHO по использованию дыхательных устройств при лечении COVID-19. Участники представляли 189 стран, 38% (6027/16 047) – страны с низким и средненизким уровнем дохода.

**Выводы:**

Привлечение добровольцев из числа инженеров по биомедицинскому оборудованию и клиницистов из стран с низким и средним уровнем дохода способствовало созданию соответствующего учебного ресурса. ВОЗ следует продолжать разработку подобных учебных пособий и предлагать их через OpenWHO, особенно в чрезвычайных ситуациях.

## Introduction

During the coronavirus disease 2019 (COVID-19) pandemic, oxygen therapy and respiratory medical technologies were vital to treat and monitor COVID-19 patients with hypoxaemia.^1^^,^^2^ As a result, large amounts of this equipment needed to be sent to countries that lacked such technology. Thus, the World Health Organization (WHO) worked to procure and deliver this life-saving equipment to such countries. By March 2021, WHO had procured 16 573 oxygen concentrators, 29 151 pulse oximeters, 2965 invasive and non-invasive patient ventilators, 4649 patient monitors and other critical clinical care supplies for shipment to 120 countries.^3^ At the same time, personnel using and maintaining the equipment needed training on these aspects to ensure their clinical effectiveness, safety and sustainability.^4^

## Local setting

OpenWHO is a free open-access platform that serves frontline responders, health workers, policy-makers and anyone wishing to learn about public health.^5^ WHO developed the platform to improve the global response to health emergencies. OpenWHO offers training courses adapted from WHO’s scientific and operational guidance. These courses are available in different languages, can be completed at the learner’s own pace and can be adapted to suit different contexts.

## Approach

In the second quarter of 2020, to provide the necessary training on the equipment, especially for resource-constrained countries, WHO assembled a team to develop a training plan, under the leadership of WHO’s medical device unit and clinical management unit. The team included WHO collaborating centres for health technology management (University of Vermont, United States of America and Pavia Hospital, Italy); International Federation for Medical and Biological Engineering, France (non-State Actor in official relations with WHO); and relevant WHO staff and consultants with no self-declared conflict of interests.

The team agreed that videos were the best media to demonstrate the use and maintenance of the equipment. The aim therefore was to produce separate training videos for the following Priority medical devices for COVID-19 treatment selected from WHO lists: continuous positive airway pressure and bilevel positive airway pressure devices; high-flow nasal cannulas; mechanical ventilators; oxygen concentrators; oxygen cylinders; patient monitors; and pulse oximeters.^2^^,^^6^

**Fig. 1:**
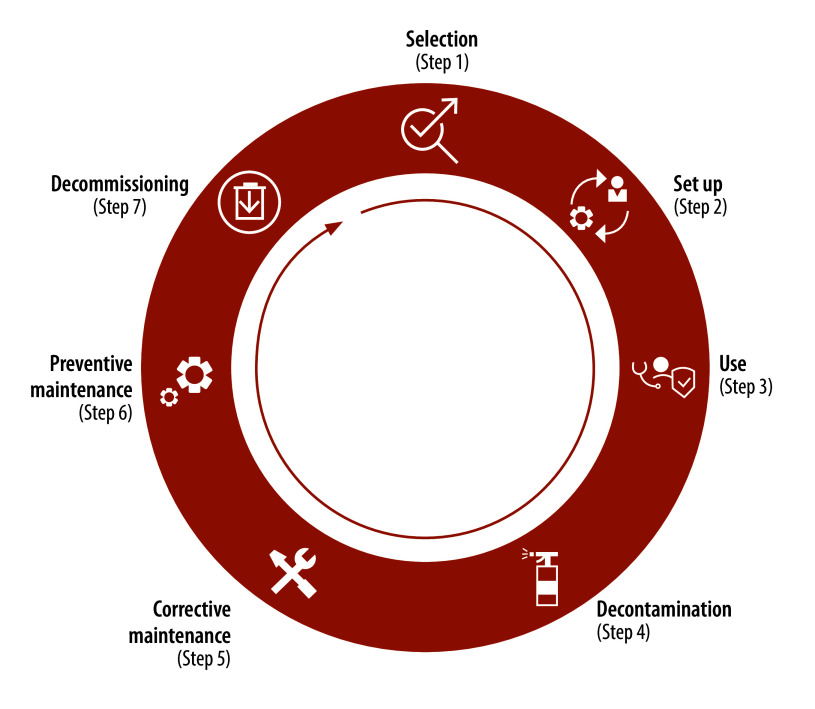
Medical equipment life cycle

Based on health technology management best practices, the videos aimed to provide information on all steps of the lifecycle of each device, namely: selection; set-up; clinical use; decontamination; preventive and corrective maintenance; and decommissioning (Fig. 1).

The Clinical Engineering Division of the International Federation for Medical and Biomedical Engineering assessed publicly available training resources to understand the existing training and the gaps in available education. They shared the information with clinical and biomedical engineering experts in the Respiratory Therapists Independent Experts Advisory Group,^7^ who provided technical input on the video scripts. 

The WHO medical devices unit and WHO staff from regional offices recruited a diverse team of biomedical engineers and physicians from various settings, including resource-constrained countries. They were invited to record preliminary training videos on their mobile telephones, after receiving the scripts and instructions from WHO. A professional editor assessed and assembled these videos into draft videos for each step and device, in line with WHO style. Voice-overs were integrated into the videos. The project team and other experts reviewed the drafts. A priority in the video development was alignment with WHO guidance and list of Priority medical devices for COVID-19 response.

On finalizing the videos, the OpenWHO COVID-19 respiratory equipment course^8^ was made available online for free.^8^ Training was complemented by YouTube videos on individual devices and lifecycle steps. WHO produced a video to publicize the release of the training courses for presentation in a webinar on 28 February 2022. The WHO medical devices newsletter, WHO Events and WHO LinkedIn posts promoted the courses, and biomedical engineer professional networks across the world were encouraged to share this information.

Due to the high demand from francophone African countries, Humatem (non-State actor in official relations with WHO) translated the videos into French, which were released on 19 September 2023 via an online seminar.^9^ The demand in eastern Europe was also high, and so the WHO Regional Office for Europe produced videos with Russian subtitles.^10^

A complementary video on electrical safety testing of medical equipment^11^ was later developed based on the International Electrotechnical Commission standard 60601–1.^12^

## Relevant changes 

The project produced 32 videos for COVID-19 respiratory equipment, with a total running time of 6 hours and 25 minutes, which were made available through OpenWHO.

As of 30 November 2023, 17 505 health workers had enrolled in the course (17 123 for the English course, 196 for the French version and 186 for the Russian version). Of these participants, 54% (5770/10 668) were male and 71% (8002/11 337) were younger than 30 years (Table 1).

**Table 1:** Features of the training courses and characteristics of course participants

Feature or characteristic	COVID-19 respiratory equipment	Medical equipment electrical safety testing
**Equipment**	Continuous and bilevel positive airway pressure devices; high-flow nasal cannulas; mechanical ventilators; oxygen concentrators; oxygen cylinders; patient monitors; and pulse oximeters	All electrical medical equipment
**Videos**		
Languages	English, French, Russian	English
No.	32	1
Total no. of videographers	64	1
Income level of videographer’s country		
Low	3	1
Lower middle	20	NA
Upper middle	12	NA
High	29	NA
**Course**		
Run time	6 h 24 min 47 s	33 min 22 s
Release date	25 Feb 2022	13 Sep 2023
Available on	OpenWHO website	OpenWHO website
Uptake	25 Feb 2022–30 Nov 2023	13 Sep 2023–4 Dec 2023
**Participants**		
No. enrolled	17 505	3 209
Age, in years, no.		
< 20	2 854	361
20–29	5 148	982
30–39	2 211	438
40–49	738	158
50–59	293	60
≥ 60	93	22
No response	6 168	1 188
Sex, no.		
Female	4 898	730
Male	5 770	1 309
No response or other	6 837	1 171
Income level of participant’s country, no.		
Low	899	364
Lower middle	5 128	1 391
Middle upper	7 439	630
High	2 581	557
No response	1 458	268
WHO region participant’s country located, no.		
African	2 418	649
Americas	1 721	230
South-East Asia	2 371	634
Europe	1 131	214
Eastern Mediterranean	2 352	775
Western Pacific	6 158	450
No response	1 354	258
**No. of countries with participants enrolled**	**189**	**137**

COVID-19: coronavirus disease 2019; NA: not applicable; WHO: World Health Organization.

The participants were from 189 countries, although 8% (1354/17 505) did not give a country. The greatest proportion of participants was from the WHO Western Pacific Region (38%; 6158/16 151), followed by 15% each from the African Region (2418/16 151) and South-East Asia Region (2371/16 151), and 14% (2352/16 151) from the Eastern Mediterranean Region. According to the World Bank Income Group Classification,^13^ 38% (6027/16 047) of the participants were from low- and lower middle-income countries (Table 1).

By the end of November 2023, 3209 people had enrolled in the Medical Equipment Electrical Safety Testing course: 64% (1309/2039) were male, and 60% (1755/2942) came from low- and lower-middle-income countries. The greatest proportion of participants was from the WHO Eastern Mediterranean Region (26%; 775/2952), followed by 22% (649/2952) from the African Region and 21% (634/2952) from the South-East Asia Region (Table 1).

The WHO medical devices training website^14^ also hosts the videos on the WHO YouTube channel to allow interested individuals access without having to enrol in the course. By March 2024, there had been 9684 viewings. The most-viewed video was *Respiratory equipment training – how to perform preventive maintenance on a patient monitor*, with 1628 views. In line with WHO YouTube channel policy, all training videos are accessible only via a link from the WHO website.

## Lessons learnt

Respiratory equipment is vital in the medical care of COVID-19 patients with hypoxia. To ensure that these medical devices can support optimal patient outcomes, training is essential in low- and middle-income countries and remote areas and should be a priority for WHO (Box 1).

Box 1 **Summary of the main lessons learnt**

- Training on the selection, use and maintenance of life-saving biomedical technology can be delivered through videos online, especially in pandemic situations.
- High-quality video training material can be co-developed with the target audience (including biomedical engineers in resource-constrained countries) together with experts if detailed instructions are provided.
- Providing training through the OpenWHO platform can reach a wide audience and will be considered by WHO for use in future training on medical devices.

WHO: World Health Organization.

The scale of the training project required input not only from technical experts; but also from users who understood training needs and would benefit from the final products. Some of the biomedical experts had no experience in making videos and primarily used mobile telephones for the videography. Thus, sufficient time was needed to prepare detailed instructions on taking videos – for example, closely following scripts, creating videos in landscape mode and having an off-screen narrator reading the script in sequence with the video.

Undertaking the activities during the COVID-19 pandemic was challenging and many delays on the original timelines occurred. The time to procure and deliver the equipment was shorter than the time needed to develop the training scripts from scratch and shoot and edit the videos. Resources for developing the training and support were also fewer than for the procurement of devices. This situation indicates that training and capacity-building are an afterthought after procurement during emergencies. Before the videos were uploaded, health workers had to learn about the equipment on the job or through information from providers or manufacturers, if available.

The video material received from the biomedical engineers and doctors in resource-constrained settings was invaluable for the team developing the training material, and enhanced interdisciplinary and cross-sectoral collaboration. These volunteer videographers disseminated information about the course widely, which undoubtedly contributed to the large enrolment numbers.

WHO funded the video production from COVID-19 emergency funds. The total cost was 100 000 United States dollars (US$) for the English version of the videos, US$ 60 000 for the French version and US$ 30 000 for coordination of both versions.

The value of the project is evident from the large number of people who have registered for the OpenWHO course across the globe – more than 17 500 people as of 30 November 2023. These participants included a large proportion from low- and lower middle-income countries, although they have the most barriers, for example, unreliable internet access. The numbers continue to grow with the addition of the French and Russian versions. This global response indicates that there is an unmet need for training material from WHO for the management of health-care technologies. 

Empowering local biomedical engineers to help develop appropriate training methods for their colleagues on the proper selection, use and maintenance of medical devices for oxygen-related respiratory equipment was key. Their involvement contributed to providing an appropriate and valued training resource. 

Since medical devices play a vital role in health care, this video training can be a model for future health technology educational projects.

The main objective of this approach was to educate health workers on the selection, use and maintenance of medical equipment in an emergency context. Given the urgency of action during the COVID-19 pandemic, the course did not address assessment of learning outcomes. The addition of such assessments and outcome measurements would add value to these methods. 
